# Antioxidant Upregulation Contributes to Improvements in Diaphragm Function Following Cervical Spinal Cord Injury and Hyperbaric Oxygen Therapy

**DOI:** 10.3390/antiox15080940

**Published:** 2026-07-29

**Authors:** Christopher C. Wendler, Prajwal P. Thakre, Kyle M. Deegan, Branden L. Nguyen, Sabhya Rana, Stephanie Lapierre-Nguyen, Jie Li, Kirstyn J. Grams, Gabriel A. Iglesias Maldonado, Amber N. Calloway, Matthew R. Nebenzahl, David D. Fuller, Ashley J. Smuder

**Affiliations:** 1 Department of Applied Physiology and Kinesiology, University of Florida, Gainesville, FL 32611, USA; cwendler@ufl.edu (C.C.W.); branden.nguyen@cuanschutz.edu (B.L.N.); stephanie.lapierre-nguyen@cuanschutz.edu (S.L.-N.); jieli@peds.ufl.edu (J.L.); amberncalloway@gmail.com (A.N.C.); yankeematt03@gmail.com (M.R.N.); dfuller@phhp.ufl.edu (D.D.F.); 2 Department of Physical Therapy, University of Florida, Gainesville, FL 32603, USA; prajwal.thakre@phhp.ufl.edu (P.P.T.); kyle.deegan@phhp.ufl.edu (K.M.D.); sabhya.rana@osumc.edu (S.R.); kirstyn.grams@phhp.ufl.edu (K.J.G.); gabriel.iglesias@phhp.ufl.edu (G.A.I.M.); 3 Department of Neuroscience, Ohio State University, Columbus, OH 43210, USA

**Keywords:** respiratory, ventilatory, superoxide dismutase, glutathione peroxidase

## Abstract

Cervical spinal cord injury directly impacts the phrenic motor neuron pool, resulting in impaired ventilation and diaphragm muscle dysfunction. Previously, we demonstrated that hyperbaric oxygen (HBO) therapy, delivered during the acute phase following SCI, can attenuate diaphragm dysfunction. However, despite the demonstrated efficacy of HBO to improve muscle function, the molecular signaling pathways regulating this effect remain unclear. These experiments test the hypothesis that the protective effects of HBO therapy are associated with upregulation of the endogenous antioxidants SOD1, SOD2 and GPX1. Adult male Sprague-Dawley rats underwent a lateral spinal contusion injury at C4 followed by 10 consecutive days of HBO (3 ATA, 100% O_2_, 1 h/day). Room air-treated spinal intact and injured rats were placed in a non-pressurized chamber for equal durations. To prevent the HBO-induced increase in antioxidant expression, antisense oligonucleotides directed towards each gene of interest were administered by intraperitoneal injection daily immediately following completion of HBO exposure (10 mg/kg/day). Saline was used as both the vehicle and placebo. Our results reveal that prevention of the HBO-induced upregulation of SOD1, SOD2 and GPX1 reduces diaphragm-specific force production compared to the control, injured HBO-treated rats. This effect occurred concomitantly with increased gene expression of the inflammatory cytokines IL-6, IL-1β and TNF-α and altered redox balance in the diaphragm. Thus, these data provide evidence that the HBO-induced increase in SOD1, SOD2 and GPX1 antioxidant expression contributes, at least in part, to the therapeutic effect of HBO to preserve diaphragm function following cervical spinal cord injury.

## 1. Introduction

Cervical spinal cord injury (cSCI) disrupts descending motor pathways that control breathing, leading to diaphragm muscle weakness and ventilatory impairment [1,2]. The diaphragm is the primary inspiratory muscle and clinical symptoms of respiratory muscle dysfunction vary depending on the degree of diaphragm paresis or paralysis. Impaired breathing and cough reflex are the most common effects of cSCI, with severe injuries requiring mechanical ventilation to maintain adequate respiratory function [3,4,5]. These pathological effects of cSCI increase the risk of respiratory infection, pneumonia and atelectasis, and significantly increase hospitalization length and healthcare costs [6,7,8]. Further, management of acute respiratory complications is of critical importance as respiratory failure is the leading cause of death in people with cSCI [9,10].

Damage to the phrenic neuromuscular system and diaphragm muscle weakness following cSCI are associated with a loss of trophic support, which is essential for maintaining effective communication between phrenic motor neurons and diaphragm muscle fibers [11,12]. Disruption to this system induces activation of pro-inflammatory pathways and increases reactive oxygen species (ROS) generation, altered redox balance, muscle protein degradation and cellular apoptosis [13,14]. Clinical strategies to regenerate the injured axons, promote neuromuscular plasticity and restore respiratory muscle function are currently limited; however, work from our group has shown that initiation of hyperbaric oxygen (HBO) therapy (3 ATA; 100% O_2_) in the acute phase following cSCI is sufficient to reduce pro-inflammatory and proteolytic signaling, preserve redox balance, and increase diaphragm force production [15,16,17].

Skeletal muscle contains a coordinated network of antioxidant enzymes that are highly adaptive to changes in cellular inflammatory and redox status and evidence suggests that increased antioxidant capacity is a principal mechanism associated with HBO-induced skeletal muscle remodeling and recovery [15,18,19]. In the diaphragm, prevention of contractile dysfunction following cSCI occurred concomitant with an HBO-induced elevation in superoxide dismutase 1 (SOD1), SOD2 and glutathione peroxidase 1 (GPX1) expression [15]. Thus, these experiments utilized antisense oligonucleotides to silence the HBO-mediated induction of each endogenous antioxidant to determine if increased levels of SOD1, SOD2 and/or GPX1 are required for HBO-induced protection of diaphragm function following cSCI.

## 2. Materials and Methods

### 2.1. Overview

**Figure 1:**
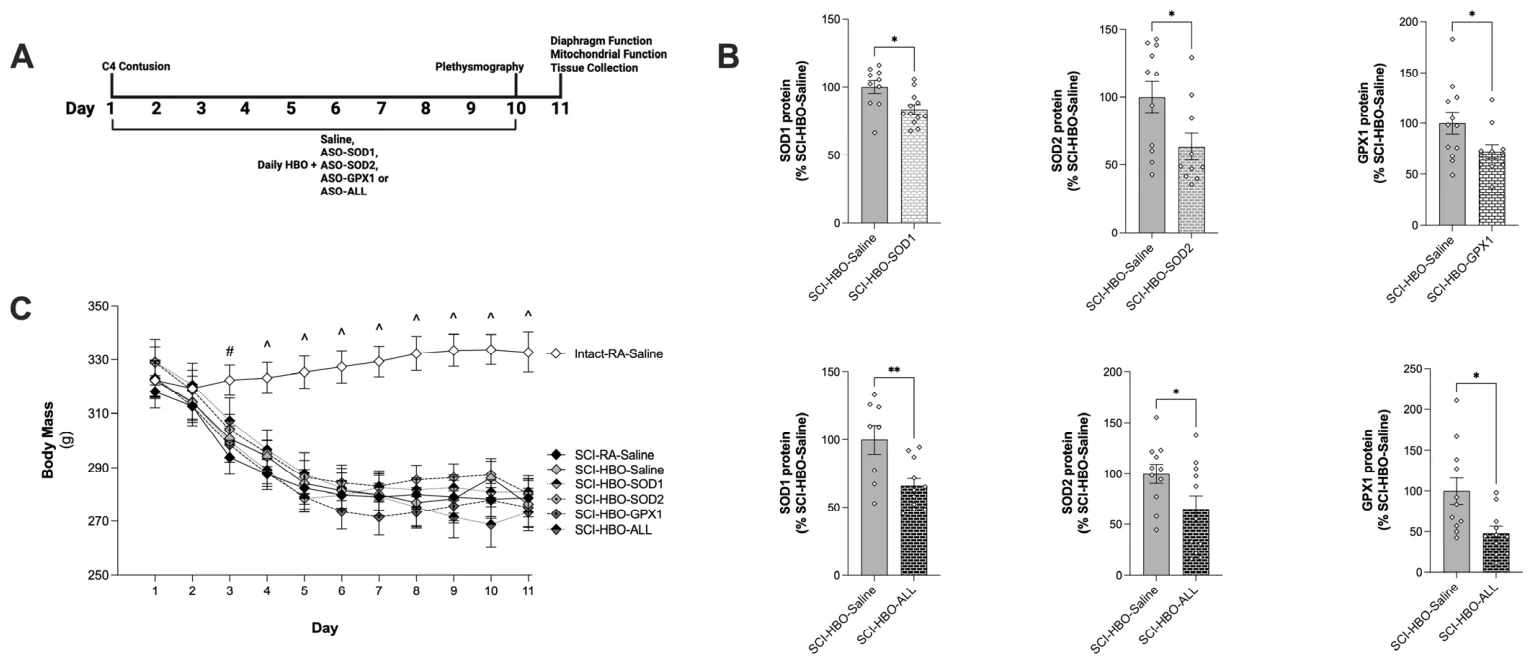
(**A**) Experimental timeline. (**B**) Western blot validation of ASO treatment. Open diamonds depict individual data points. * = *p* < 0.05 and ** = *p* < 0.01. (**C**) Change in body mass over the experimental timeline. # = significantly different vs. SCI-RA-Saline, ^ = significantly different vs. SCI-RA-Saline and SCI-HBO-Saline. Values are presented as mean ± SEM. Group sample sizes: (**B**) SCI-HBO-saline, *n* = 8–12; SCI-HBO-SOD1, *n* = 12; SCI-HBO-SOD2, *n* = 10; SCI-HBO-GPX1, *n* = 11; SCI-HBO-ALL, *n* = 11. Group sample sizes (**C**): *n* = 12 (Intact-RA-Saline, SCI-HBO-Saline and SCI-HBO-SOD1) and *n* = 11 (SCI-RA-Saline, SCI-HBO-SOD2, SCI-HBO-GPX1 and SCI-HBO-ALL).

Adult (3-month-old) male Sprague-Dawley rats were obtained from Envigo (Indianapolis, IN, USA) and housed at the McKnight Brain Institute Animal Care Facility at the University of Florida. The University of Florida Institutional Animal Care and Use Committee approved these experiments and they were performed in accordance with NIH guidelines. All rats were housed identically and had tunnels and nylon bones for environmental enrichment. Rats were randomly separated into seven groups using an online random number generator. One group served as a spinal intact control group and was exposed to 10 consecutive days of treatment with room air (Intact-RA-Saline; *n* = 12). One group had a cSCI and was exposed to 10 consecutive days of treatment with room air (SCI-RA-Saline; *n* = 11). The remaining five groups had a cSCI and were exposed to 10 consecutive days of HBO therapy and received saline (SCI-HBO-Saline; *n* = 12) or an antisense oligonucleotide(s) (ASO) targeting superoxide dismutase 1 (SOD1) (SCI-HBO-SOD1; *n* = 12), superoxide dismutase 2 (SOD2) (SCI-HBO-SOD2; *n* = 11) or glutathione peroxidase 1 (GPX1) (SCI-HBO-GPX1; *n* = 11). The SCI-HBO-ALL (*n* = 11) group received a combination of all three ASOs. The experimental design is depicted in Figure 1A. Where sample size was reduced, rats that exhibited forelimb autotomy were euthanized in accordance with IACUC humane endpoints. In addition, two rats developed sepsis and were excluded from the study (*n* = 1; SCI-RA-Saline and SCI-HBO-SOD2). All treatments, care and measures were performed identically and by blinded researchers to reduce potential confounders.

### 2.2. Cervical Spinal Cord Contusion

All injuries were performed as previously described [16,20]. Briefly, male rats were anesthetized with ketamine (100 mg/kg) and xylazine (10 mg/kg, i.p.). The musculature overlying C2-C6 was exposed and retracted to allow visualization of the cervical vertebrae. The vertebrae were cleared of surrounding tissue using a curette. Animals were stabilized with clamps at C3 and C5 and underwent a laminectomy at the fourth cervical (C4) vertebra. A unilateral cervical spinal cord contusion was induced using an Infinite Horizons Impactor (2.5 mm tip; Precision Systems and Instrumentation, Lexington, KY, USA) delivering a 150 kdyn force to the right side of the spinal cord. Overlying musculature was sutured using sterile 4-0 Vicryl suture and 9 mm wound clips were used to close the skin. For post-operative analgesia, animals received a single subcutaneous injection of extended-release buprenorphine (Buprenorphine SR-LAB, 1 mg/kg), followed by a 48 h post-operative regimen consisting of carprofen (5 mg/kg, q.d.), enrofloxacin (Baytril, 5 mg/kg, q.d.), and lactated Ringer’s solution (10 mL/kg, q.d.) for fluid support. Animals were assessed daily for behavioral and physical indicators of distress, including dehydration and body weight changes.

### 2.3. HBO Therapy and Antisense Oligonucleotide Administration

HBO therapy was initiated on the day of cSCI and continued for 10 consecutive days. Rats were placed within a custom 32 L hyperbaric research chamber (PCCI Hyperbaric Systems, Alexandria, VA, USA) and were exposed to 100% O_2_ at 3 ATA for one hour. During HBO exposure, the chamber was continuously flushed with O_2_ to prevent CO_2_ buildup. Immediately following each HBO exposure, rats received an i.p. injection of their designated ASO phosphorothioate (Integrated DNA Technologies, Coralville, IA, USA) at a dose of 10 mg/kg. The SCI-HBO-ALL group received 10 mg/kg/day of each ASO. The dose was chosen based on previous work by our group using ASOs to knockdown protein expression in the diaphragm [21,22,23]. The following sequences were designed to specifically target SOD1, SOD2 and GPX1 to inhibit protein translation:

SOD1: 5′-CACGGCCTTCATCGCCATGCTTC-3′;

SOD2: 5′-CACGCCGCCCGACACAACATTG-3′;

GPX1: 5′-GCCTCAGAGGGACGCGACATTCTCA-3′.

Saline was used as the vehicle and as the placebo. Rats were randomly assigned to a group and treatments were coded to blind researchers. Rats breathing room air were exposed to the non-pressurized chamber for an equal duration as the HBO-treated rats. Following the final HBO or room air exposure, ventilation was measured with a whole-body plethysmograph and 24 h later, plasma was collected and the diaphragm was removed for subsequent analyses. All dependent measures were performed on the hemi-diaphragm ipsilateral to the contusion.

### 2.4. Whole Body Plethysmography

Overall ventilation was measured using flow-through whole body plethysmography in awake, freely moving male rats [24]. Rats were placed in standard plethysmography chambers (Buxco, Wilmington, NC, USA) with a continuous flow rate of 6 L/min. Chamber CO_2_ concentrations (outflow), temperature, humidity and chamber pressure were measured continuously. The experimental protocol consisted of a 40 min normoxic baseline period (“Baseline”, FIO_2_ = 0.21; N_2_ balance) followed by a 7 min period of hypoxic-hypercapnic challenge (“Max Chemo”, FIO_2_ = 0.10; FICO_2_ = 0.07, N_2_ balance). Rectal temperature was recorded immediately before and after each session. Missing data points were due to equipment malfunction. Investigators were blinded to the experimental groups during data collection and analysis.

### 2.5. Plethysmography Waveform Data Analysis and Processing

Respiratory signals were sampled at 500 Hz using Spike2 software v10.01. For analysis, ~5 min of stable eupneic breathing during the normoxic baseline period were selected. Tidal volume was calculated from the filtered flow signal using a calibration factor and corrected for chamber temperature and humidity using the Drorbaugh–Fenn equation [25].

To assess tidal volume usage patterns, waveform analysis was performed using custom MATLAB R2024a scripts adapted from previously published methods [26]. Approximately 20 min of baseline data were bandpass filtered (second-order Butterworth, 0.5–10 Hz) and downsampled to 90 Hz. Individual breaths were identified using a threshold crossing method to detect inspiratory peaks, with inspiratory onset defined by rising zero crossings. Breath detection windows were defined by shifting the waveform window by ~20% of the total breath duration, which was initially bound by the subsequent inspiratory onset. Tidal volume was calculated for each breath using the Drorbaugh–Fenn equation [25]. Breath waveforms were then grouped into predefined categories based on total breath duration (Ttot): 0.0–0.2 s, 0.2–0.5 s, 0.5–1.2 s, and 1.2–4.0 s. Within each category, principal component analysis was applied for dimensional reduction, followed by hierarchical clustering. The optimal number of clusters was determined using an elbow method. Mean tidal volume was calculated for each cluster and each animal’s proportional usage of clusters was used to compute a weighted average tidal volume. Clusters were subsequently categorized into low-, mid-, and high-tidal volume groups based on tertile threshold. The proportion of breaths within each category was then calculated for each animal. For the clustering analysis, data pooled from breath waveforms from all rats were used. This allowed the same waveform clusters to be applied across rats and treatment groups. To avoid pseudoreplication, individual breaths were not treated as independent samples. Differences in the total number of breaths across animals were accounted for by calculating the percentage of breaths assigned to each cluster for each animal. Group comparisons were then performed using these animal-level values.

Inspiratory time (TI) and expiratory time (TE) were quantified based on durations of inhalation and expiration, respectively. Inspiratory onset and offset were determined using rising zero crossing and peak inspiratory flow, respectively. Breathing variability was assessed using the Poincaré method of total breath duration (Ttot) [27]. Variability was quantified by dispersion along the line of identity (SD1, short-term variability) and perpendicular to the dispersion (SD2, long-term variability), corresponding to length and the width of the fitted ellipse, respectively.

### 2.6. Diaphragm Contractile Function

A strip of medial costal diaphragm muscle from the ipsilateral diaphragm was suspended vertically between two platinum electrodes in a jacketed tissue bath containing Krebs–Henseleit buffer (Sigma-Aldrich, St. Louis, MO, USA) continuously infused with 95% O_2_ and 5% CO_2_ [16]. Optimal length (L_o_) of the muscle was determined and the force-frequency response was measured over a range of 1, 15, 30, 60, 100 and 160 Hz. Isometric contractile properties of the diaphragm were measured at twitch (1 Hz) stimulation. The force output was normalized to total muscle cross-sectional area and reported as specific force (N/cm^2^). Investigators were blinded to the experimental groups. Diaphragms damaged during dissection were removed from the analysis.

### 2.7. Diaphragm Cross-Sectional Area and Fiber Type

A strip of medial costal diaphragm collected from the ipsilateral diaphragm was frozen in OCT in liquid nitrogen-cooled isopentane [16]. Diaphragm sections damaged during freezing were removed from the study. Ten-micron cross-sections were cut using a cryostat (Leica CM 3050 S, Leica Biosystems, Deer Park, IL, USA) and stained with primary antibodies against laminin (L9393, Sigma-Aldrich), myosin heavy chain type I (A4.840) and myosin heavy chain type IIa (SC-71) (Developmental Studies Hybridoma Bank, Iowa City, IA, USA). Diaphragm cross-sections were imaged using a Revolve fluorescent microscope (Echo, San Diego, CA, USA). Fiber cross-sectional area and fiber type were determined using ImageJ software v1.52k (NIH, Bethesda, MD, USA). Investigators were blinded to the experimental groups and a minimum of 250 independent fibers were analyzed for each diaphragm [28].

### 2.8. Mitochondrial Respiration and H_2_O_2_ Production

An Oroboros O2k high-resolution respirometer (Oroboros, Innsbruck, Austria) was used to assess mitochondrial oxygen consumption in permeabilized diaphragm muscle fibers. Fiber bundles were teased apart in ice-cold Buffer X (in mM: 60 K-MES, 35 KCl, 7.23 K_2_EGTA, 2.77 CaK_2_EGTA, 20 imidazole, 0.5 dithiothreitol, 20 taurine, 5.7 ATP, 15 phosphocreatine, and 6.56 MgCl_2_; pH 7.1), permeabilized in Buffer X containing 50 μg/mL saponin, then washed for 15 min in Buffer D (in mM 105 KMES, 30 KCl, 1 EGTA, 10 KH_2_PO_4_, 5 MgCl_2_-6H_2_O, 0.05% BSA, pH 7.1). To measure respiration, 2.1 mL of Buffer D, 5mM creatine monohydrate and the fiber bundle were added to the respiration chamber. State 2 respiration was initiated with 20 mM creatine kinase with complex I substrates 2.5 mM malate and 5 mM pyruvate. Maximal *J*O_2_ (State 3) was driven with the addition of 1 mM phosphocreatine (PCr) and 5 mM ATP. Cytochrome C (0.005 mM) was used to assess mitochondrial integrity, with a 15% increase in respiration the threshold for exclusion of replicates. Additional data points were excluded due to tissue loss. Sequential PCr titrations were then added at 3 mM, 6 mM, 15 mM and 30 mM. Calculations of ∆G_ATP_ were performed using an online calculator [29]. H_2_O_2_ emission in permeabilized diaphragm muscle fibers was determined using Amplex Red (Molecular Probes, Eugene, OR, USA) and a SpectraMax M5 spectrophotometer (Molecular Devices, San Jose, CA, USA) as described [15]. This assay uses horseradish peroxidase to catalyze the H_2_O_2_-dependent oxidation of Amplex Red to resorufin. H_2_O_2_ production was calculated using a standard curve and is presented as the H_2_O_2_ emission over ten minutes normalized to the dry weight of the fiber bundle (pmol/min/mg dry weight) [21]. Excluded data points were due to errors in acquiring the standard curve. Respiration and H_2_O_2_ production measurements were made in duplicate and investigators were blinded to the experimental groups.

### 2.9. RT-PCR

Total RNA from the diaphragm was isolated using a Qiagen RNeasy kit (Hilden, Germany); quality was confirmed by evaluating the 260/280 and 260/230 ratios and 0.5 µg RNA was reverse transcribed with the Superscript III First-Strand Synthesis System for RT-PCR (ThermoFisher Scientific, Waltham, MA, USA). A total of 1 μL of cDNA was added to a 24 µL PCR reaction for RT-PCR using Taqman chemistry and the StepOnePlus RT-PCR system (ThermoFisher Scientific). Relative quantification of gene expression was performed using the 2^−ΔΔCt^ method. Interleukin (IL) -1β (IL-1β) (Rn00580432_m1), IL-6 (Rn01410330_m1), tumor necrosis factor alpha (TNF-α) (Rn99999017_m1), MaFbx/atrogin-1 (Rn00591730_m1) and MuRF1 (Rn00590197_m1) were assayed using predesigned rat primer and probe sequences commercially available from ThermoFisher Scientific. Samples were run in duplicate and the average of the replicates was used for analysis. Replicates that failed to amplify were excluded from the analysis. β-glucuronidase (Rn00566655_m1) was chosen as the reference gene based on previous work showing unchanged expression in the diaphragm with cSCI, HBO and/or ASO treatment [11,15,21] and its stable expression in the diaphragm of our treatment groups.

### 2.10. Western Blot Analysis

Western blotting was used to validate the efficacy of each ASO to decrease protein expression of the targeted protein compared to the SCI-HBO-Saline group. Western blot analysis was performed based on previous evidence from our group demonstrating increased diaphragm protein levels of SOD1, SOD2 and GPX1 in cSCI rats treated with HBO compared to room air [15]. Diaphragm muscle was homogenized 1:10 in Tris-EDTA buffer with a protease inhibitor cocktail and protein concentrations were normalized using the Bradford method. Proteins were run on 4–20% precast gels (Bio-Rad Laboratories, Hercules, CA, USA), transferred to nitrocellulose membrane and total protein was imaged following incubation with Revert^®^ Total Protein Stain (LI-COR, Lincoln, NE, USA). Total protein stain was removed and membranes were blocked in 5% non-fat milk followed by incubation with primary antibodies targeting SOD1 (10269-1-AP, Proteintech, Rosemont, IL, USA), SOD2 (ab68155, Abcam, Waltham, MA, USA) and GPX1 (ab22604, Abcam). Antibodies are all knockout validated and were previously tested by serial dilution to determine their linear range. Membranes were imaged with an Odyssey CLx (LI-COR) and analyzed using Image Studio software v6.1.0.79 (LI-COR). Protein abundance was normalized to total protein levels. One sample was removed from analysis where loading volume was inadvertently reduced compared to other samples.

### 2.11. Plasma Analysis

Blood was collected, placed in K_3_EDTA-coated tubes and centrifuged at 5000 rpm for 10 min at 4 °C. Plasma was assayed using a Luminex MAGPIX^®^ multiplex analyzer (Millipore Sigma, Burlington, MA, USA). Plasma cytokines and myokines were assayed in duplicate with the 27-Plex MILLIPLEX^®^ MAP Rat Cytokine/Chemokine Magnetic Bead Panel (RECYTMAG-65K) and the 12-Plex MILLIPLEX Rat Myokine Panel (RMYOMAG-88K) according to manufacturer’s instructions. Investigators were blinded to the experimental groups. Missing data points were due to lack of sufficient plasma volume. Analysis was performed using Belysa^®^ Immunoassay Curve Fitting Software v1.2 (Millipore Sigma).

### 2.12. Statistical Analysis

Data are presented as mean ± standard error of the mean (SEM) with individual data points depicted to illustrate group deviation. Sample size was determined using power calculations based on effect sizes measured for cSCI and HBO on diaphragm muscle force production measured in our previous publications [15,16]. This calculation determined *n* = 11/group was required, with a sample size of *n* = 12 used to account for unexpected loss in an experimental group. For each dependent measure, a Shapiro–Wilk test was used to test for normal distribution and an F-test or Bartlett’s test was used to compare variances between groups. To test for differences between the Intact-RA-Saline, SCI-RA-Saline and SCI-HBO-Saline groups, parametric data was evaluated using a one-way analysis of variance (ANOVA) followed by Tukey’s post hoc test when applicable. Nonparametric data was analyzed using the Kruskal–Wallis test, followed by Dunn’s post hoc test when appropriate. For parametric data with unequal variances, Brown–Forsythe and Welch ANOVAs were performed, followed by a Dunnett T3 test when appropriate. To test for differences between the SCI-HBO-Saline group and the SCI-HBO-SOD1, SCI-HBO-SOD2, SCI-HBO-GPX1 or SCI-HBO-ALL group, parametric data were evaluated using a one-way ANOVA followed by a Dunnett’s post hoc test. Nonparametric data was analyzed using the Kruskal–Wallis test followed by Dunn’s post hoc test when appropriate and parametric data with unequal variances were analyzed by Brown–Forsythe and Welch ANOVAs followed by a Dunnett T3 test when appropriate. To assess changes in body mass over the experimental timeline, a two-way repeated measures ANOVA was used to determine whether main effects of experiment day or body mass or an interaction existed between the Intact-RA-Saline, SCI-RA-Saline and SCI-HBO-Saline groups. When there was a significant interaction, Tukey’s post hoc test was performed to evaluate significant differences in body mass between groups at each experimental day. Due to a missing data point in the SCI-HBO-SOD2 group, a mixed effects model and a Dunnett post hoc test were used to test for differences between the SCI-HBO-Saline group and the SCI-HBO-SOD1, SCI-HBO-SOD2, SCI-HBO-GPX1 or SCI-HBO-ALL group at each experimental day. To analyze the force frequency response, a two-way repeated measures ANOVA was used to determine whether main effects of frequency or specific force or an interaction existed between groups. When applicable, Tukey’s post hoc test was performed to evaluate significant differences in specific force between the Intact-RA-Saline, SCI-RA-Saline and SCI-HBO-Saline groups at each frequency or a Dunnett’s post hoc test was used to test for differences in specific force between the SCI-HBO-Saline group and the SCI-HBO-SOD1, SCI-HBO-SOD2, SCI-HBO-GPX1 or SCI-HBO-ALL group at each frequency. A Geisser–Greenhouse correction was performed when necessary to correct for sphericity. Plasma data underwent a square root transformation prior to analysis and the Benjamini, Krieger and Yekutieli test was used to control for False Discovery Rate. Statistical analyses were performed using GraphPad Prism 10 software and significance was established at *p* < 0.05.

## 3. Results

### 3.1. Treatment Validation

Efficacy of each ASO to reduce diaphragm expression of the targeted protein was confirmed by Western blotting (Figure 1B). Our results show a significant decrease in antioxidant expression corresponding to the ASO(s) administered when compared to the saline-treated SCI-HBO control group.

### 3.2. Animal Body Mass

No difference in body mass existed between groups prior to initiating the experimental protocols (Day 1; Figure 1C). Following cSCI, rats treated with room air had significantly reduced body mass compared to the spinal-intact rats starting on day 3 and continuing until the experimental endpoint on day 11. cSCI rats treated with HBO had significantly reduced body mass compared to spinal-intact rats starting at day 4, continuing through day 11. Body mass was not different between the SCI-HBO-Saline group and the ASO-treated groups at any time (Figure 1C).

### 3.3. Respiratory Function

**Figure 2:**
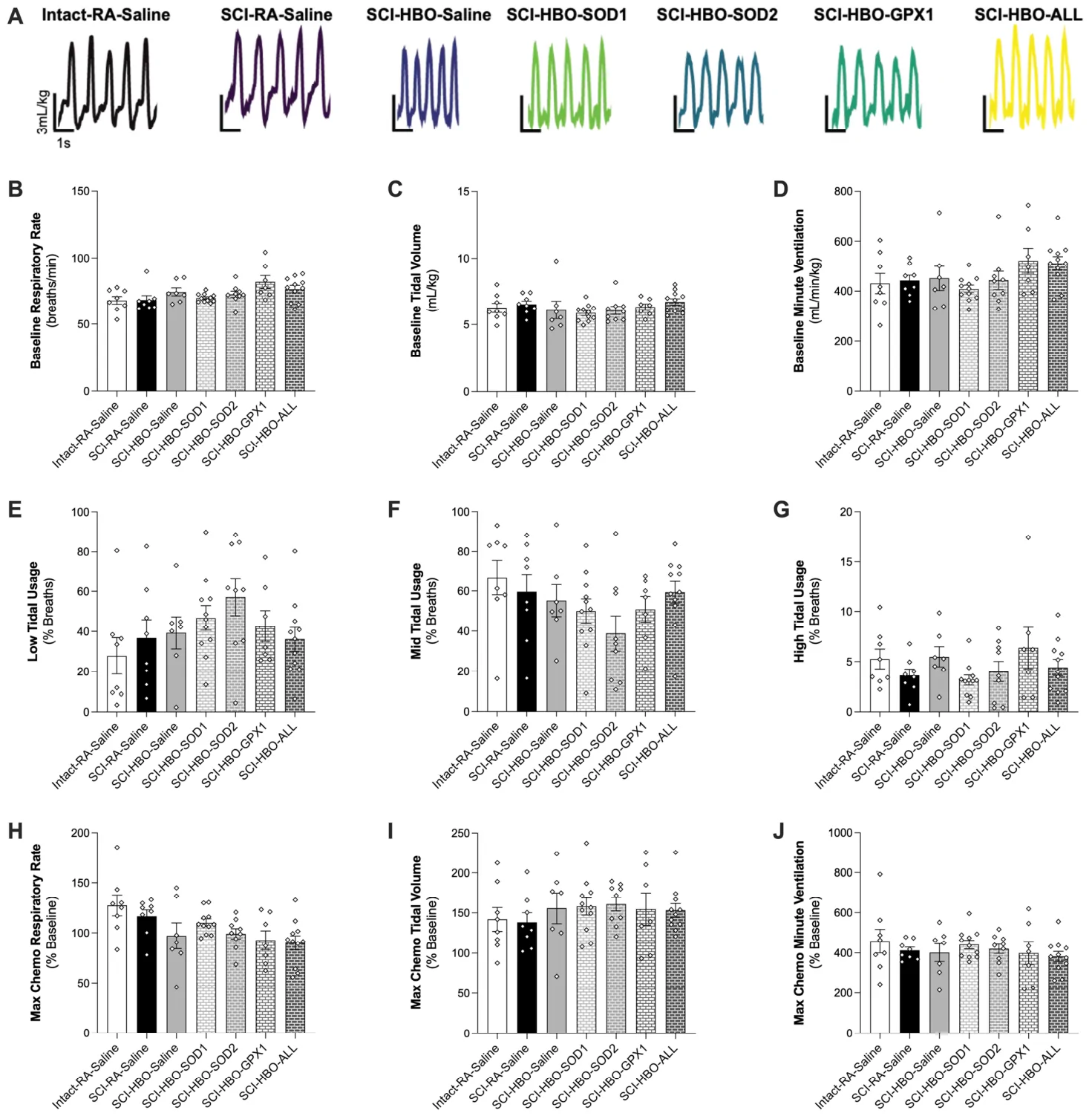
(**A**) Representative baseline respiratory tracing for each experimental group; (**B**) respiratory rate; (**C**) tidal volume; and (**D**) minute ventilation during baseline eupneic breathing. Percentage of (**E**) low-; (**F**) mid-; and (**G**) high-tidal usage during a period of five minutes of eupneic breathing. Percentage change in (**H**) respiratory rate; (**I**) tidal volume; and (**J**) minute ventilation during a Max Chemo challenge compared to baseline. Open diamonds depict individual data points. Values are presented as mean ± SEM. Group sample sizes: *n* = 7 (SCI-HBO-Saline and SCI-HBO-GPX1); *n* = 8 (Intact-RA-Saline and SCI-RA-Saline); *n* = 9 (SCI-HBO-SOD2); and *n* = 11 (SCI-HBO-SOD1 and SCI-HBO-ALL).

**Figure 3:**
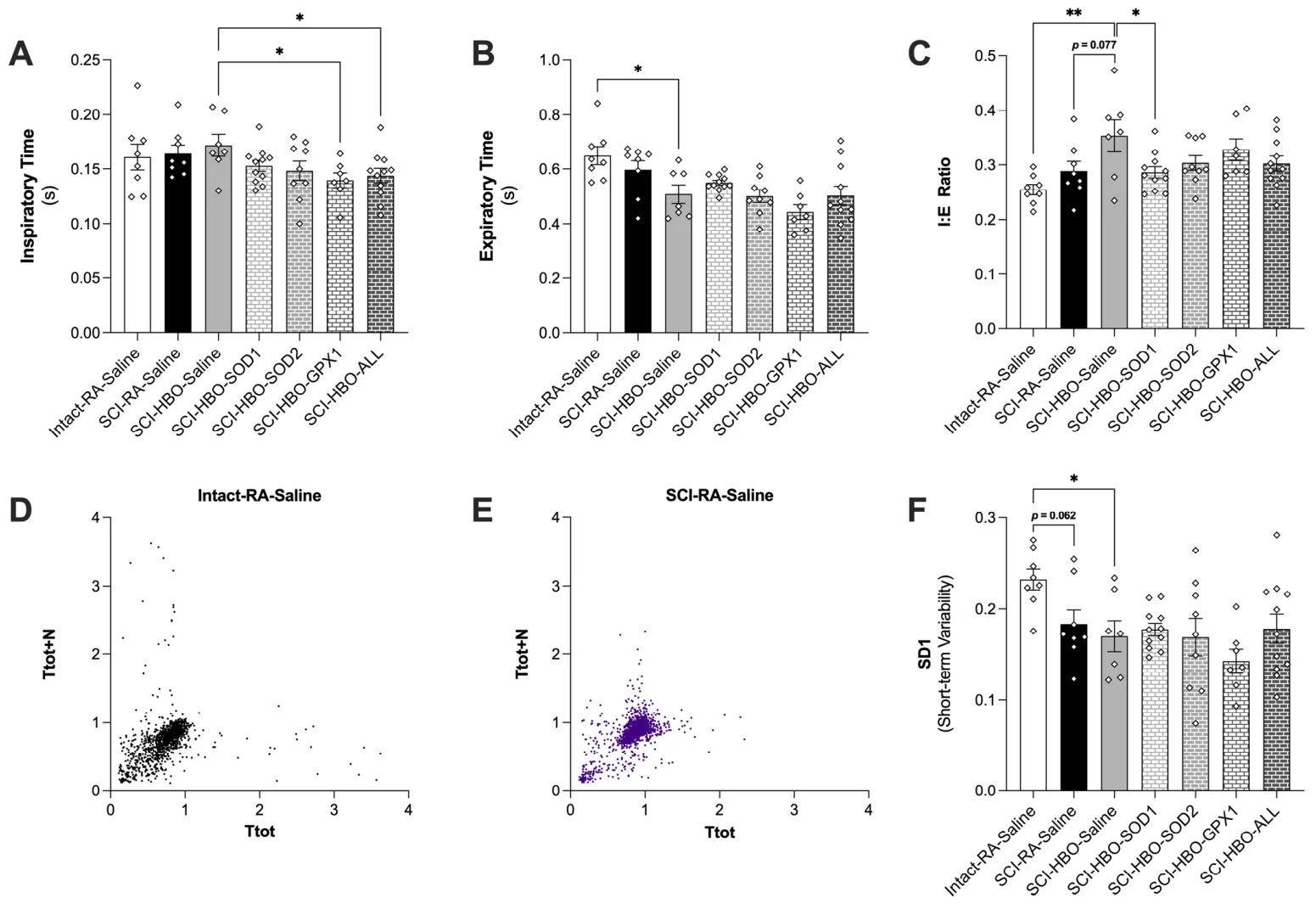
Breath (**A**) inspiratory time; (**B**) expiratory time; and (**C**) ratio of inspiratory to expiratory time (I:E ratio). Poincaré plot demonstrating breathing variability for the (**D**) Intact-RA-Saline group and the (**E**) SCI-RA-Saline group. (**F**) SD1 demonstrating short-term breathing variability. Open diamonds depict individual data points. Values are presented as mean ± SEM. * = *p* < 0.05 and ** = *p* < 0.01. Group sample sizes: *n* = 7 (SCI-HBO-Saline and SCI-HBO-GPX1); *n* = 8 (Intact-RA-Saline and SCI-RA-Saline); *n* = 9 (SCI-HBO-SOD2); and *n* = 11 (SCI-HBO-SOD1 and SCI-HBO-ALL).

Evaluation of baseline ventilation between groups showed no difference in respiratory rate, tidal volume or minute ventilation between groups (Figure 2A–D). In addition, there were no differences in the percentage of low-, mid- or high-tidal usage breaths (Figure 2E–G). There was also no difference between groups when evaluating the percent change in respiratory rate, tidal volume or minute ventilation during the Max Chemo challenge compared to baseline respiration (Figure 2H–J). For each breath, inspiratory time was significantly greater in the SCI-HBO-Saline group compared to the SCI-HBO-GPX1 and SCI-HBO-ALL groups (Figure 3A) and expiratory time was reduced in the SCI-HBO-Saline group compared to the Intact-RA-Saline group (Figure 3B). The ratio of inspiratory to expiratory time (I:E ratio) was elevated in the SCI-HBO-Saline group compared to the Intact-RA-Saline and SCI-HBO-SOD1 groups, with a trend for the I:E ratio to also be elevated compared to the SCI-RA-Saline group (*p* = 0.077) (Figure 3C). Finally, measurement of total breath duration revealed a reduction in SD1 in the SCI-HBO-Saline group and the SCI-RA-Saline group (*p* = 0.062) compared to the Intact-RA-Saline group (Figure 3D–F).

### 3.4. Diaphragm Force Production and CSA

**Figure 4:**
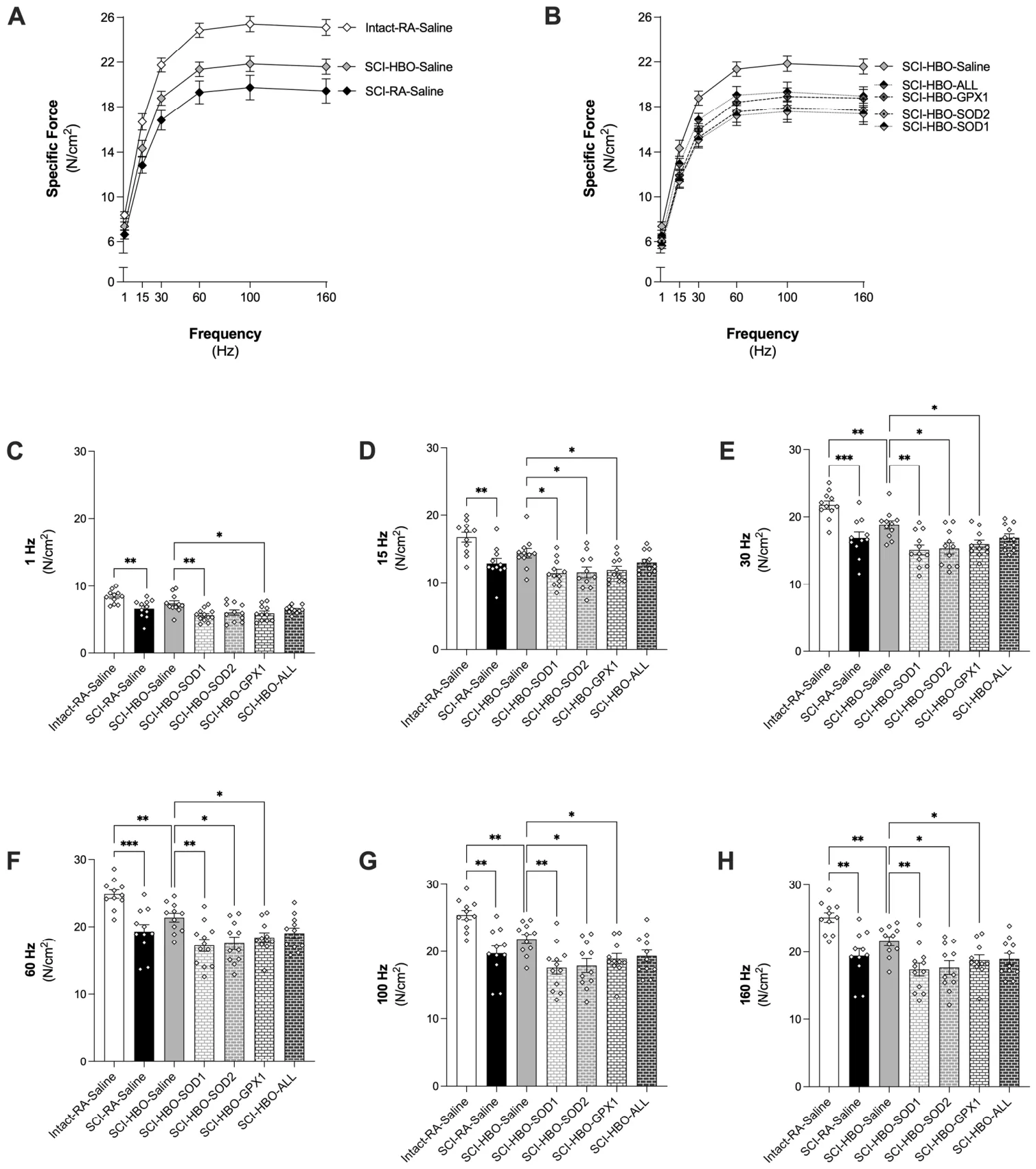
Diaphragm muscle (**A**) force frequency response for the Intact-RA-Saline, SCI-RA-Saline and SCI-HBO-Saline groups and (**B**) force frequency response for the SCI-HBO-Saline, SCI-HBO-SOD1, SCI-HBO-SOD2, SCI-HBO-GPX1 and SCI-HBO-ALL groups. Specific force at (**C**) 1 Hz; (**D**) 15 Hz; (**E**) 30 Hz; (**F**) 60 Hz; (**G**) 100 Hz and (**H**) 160 Hz. Open diamonds depict individual data points. Values are presented as mean ± SEM. * = *p* < 0.05, ** = *p* < 0.01 and *** = *p* < 0.001. Group sample sizes: *n* = 12 (SCI-HBO-SOD1); and *n* = 11 (Intact-RA-Saline, SCI-RA-Saline, SCI-HBO-Saline, SCI-HBO-SOD2, SCI-HBO-GPX1 and SCI-HBO-ALL).

**Figure 5:**
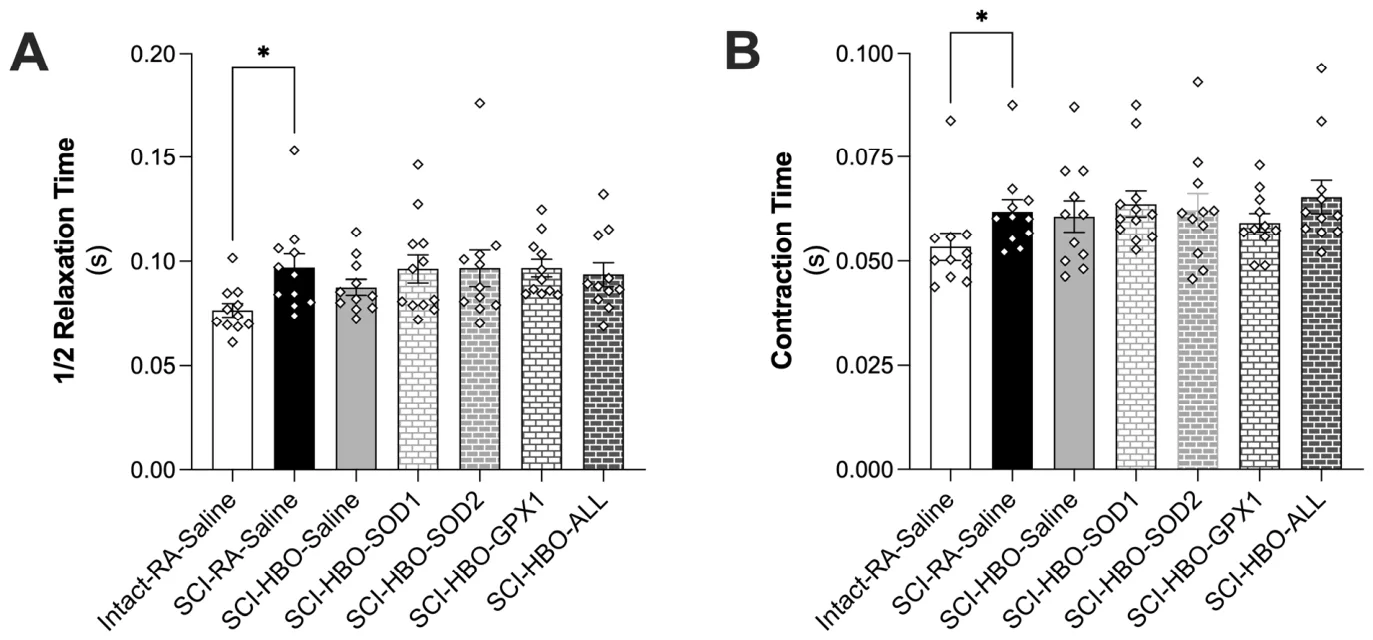
Diaphragm muscle twitch force (**A**) time to ½ relaxation and (**B**) time to max contraction. Open diamonds depict individual data points. Values are presented as mean ± SEM. * = *p* < 0.05. Group sample sizes: *n* = 12 (SCI-HBO-SOD1); and *n* = 11 (Intact-RA-Saline, SCI-RA-Saline, SCI-HBO-Saline, SCI-HBO-SOD2, SCI-HBO-GPX1 and SCI-HBO-ALL).

**Figure 6:**
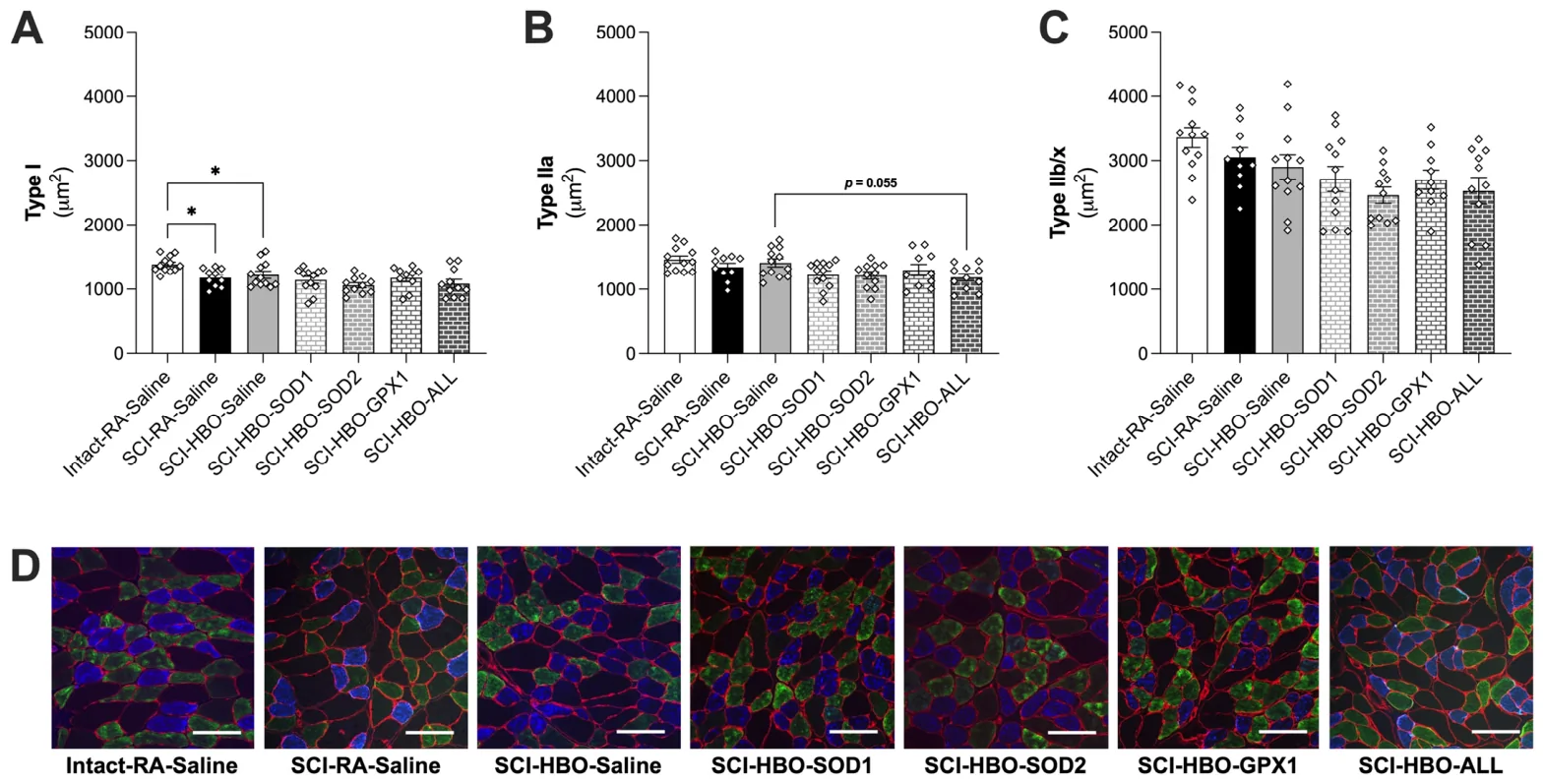
Diaphragm muscle cross-sectional area for (**A**) type I; (**B**) type IIa and (**C**) type IIb/x fibers. (**D**) Representative diaphragm cross-sections. Scale bar = 100 μm. Open diamonds depict individual data points. Values are presented as mean ± SEM. * = *p* < 0.05. Group sample sizes: *n* = 12 (Intact-RA-Saline, SCI-HBO-Saline and SCI-HBO-SOD1); *n* = 11 (SCI-HBO-SOD2 and SCI-HBO-ALL); and *n* = 10 (SCI-RA-Saline and SCI-HBO-GPX1).

Comparison of diaphragm muscle-specific force production between the Intact-RA-Saline, SCI-RA-Saline and SCI-HBO-Saline groups showed a significant reduction in force in the SCI-RA-Saline group compared to the Intact-RA-Saline group at all frequencies tested (1–160 Hz) (Figure 4A–H). Specific force was reduced in the SCI-HBO-Saline group for frequencies 30 Hz and greater. Compared to SCI-HBO-Saline, independent knockdown of SOD1 or GPX1 resulted in a reduction in diaphragm specific force production at frequencies of 1–160 Hz and knockdown of SOD2 resulted in lower force production at frequencies of 15–160 Hz. Twitch force characteristics show a significant increase in the ½ RT and time to maximum force in the SCI-RA-Saline group compared to the Intact-RA-Saline group (Figure 5A,B). Diaphragm muscle type I fiber CSA was significantly lower in both the SCI-RA-Saline and SCI-HBO-Saline groups compared to Intact-RA-Saline (Figure 6A). There was a trend for type IIa fibers in the SCI-HBO-ALL group to be smaller than the SCI-HBO-Saline group (*p* = 0.055). No other differences in diaphragm muscle fiber CSA were seen.

### 3.5. Diaphragm Mitochondrial Respiration and H_2_O_2_ Production

**Figure 7:**
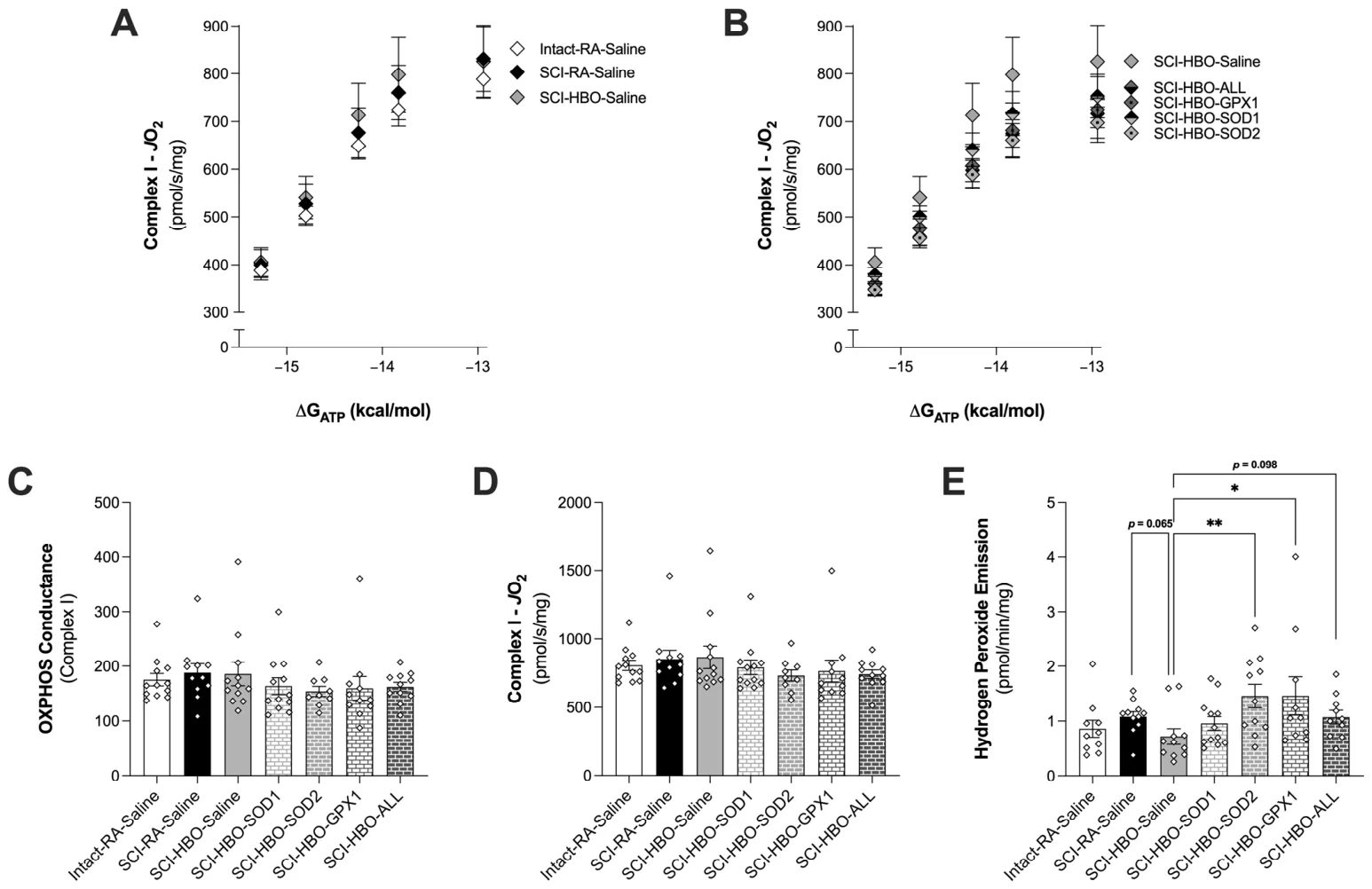
Diaphragm muscle mitochondrial oxygen consumption and H_2_O_2_ emission in permeabilized muscle fiber bundles. Mitochondrial OXPHOS in the (**A**) Intact-RA-Saline, SCI-RA-Saline and SCI-HBO-Saline groups and the (**B**) SCI-HBO-Saline, SCI-HBO-SOD1, SCI-HBO-SOD2, SCI-HBO-GPX1 and SCI-HBO-ALL groups. (**C**) Quantification of OXPHOS conductance. (**D**) Maximal ADP-stimulated respiration supported by pyruvate and malate. (**E**) Diaphragm mitochondrial H_2_O_2_ emission. Open diamonds depict individual data points. Values are presented as mean ± SEM. * = *p* < 0.05 and ** = *p* < 0.01. Group sample sizes for (**A**–**D**): *n* = 12 (Intact-RA-Saline, SCI-HBO-Saline and SCI-HBO-SOD1); *n* = 11 (SCI-RA-Saline, SCI-HBO-GPX1 and SCI-HBO-ALL); and *n* = 9 (and SCI-HBO-SOD2). Group sample sizes for (**E**): *n* = 12 (SCI-HBO-SOD1); *n* = 11 (SCI-RA-Saline, SCI-HBO-Saline and SCI-HBO-SOD2); and *n* = 10 (Intact-RA-Saline, SCI-HBO-GPX1 and SCI-HBO-ALL).

Measurement of mitochondrial complex I maximal rate of oxygen consumption and OXPHOS conductance showed no difference between groups (Figure 7A–D). There was a trend for HBO therapy to reduce mitochondrial H_2_O_2_ production in the diaphragm compared to RA-exposed rats (*p* = 0.065). Diaphragm mitochondrial H_2_O_2_ emission was significantly higher in the SCI-HBO-SOD2 and SCI-HBO-GPX1 groups, with *p* = 0.098 when comparing the SCI-HBO-ALL group with the SCI-HBO-Saline group (Figure 7E).

### 3.6. Diaphragm Gene Expression

**Figure 8:**
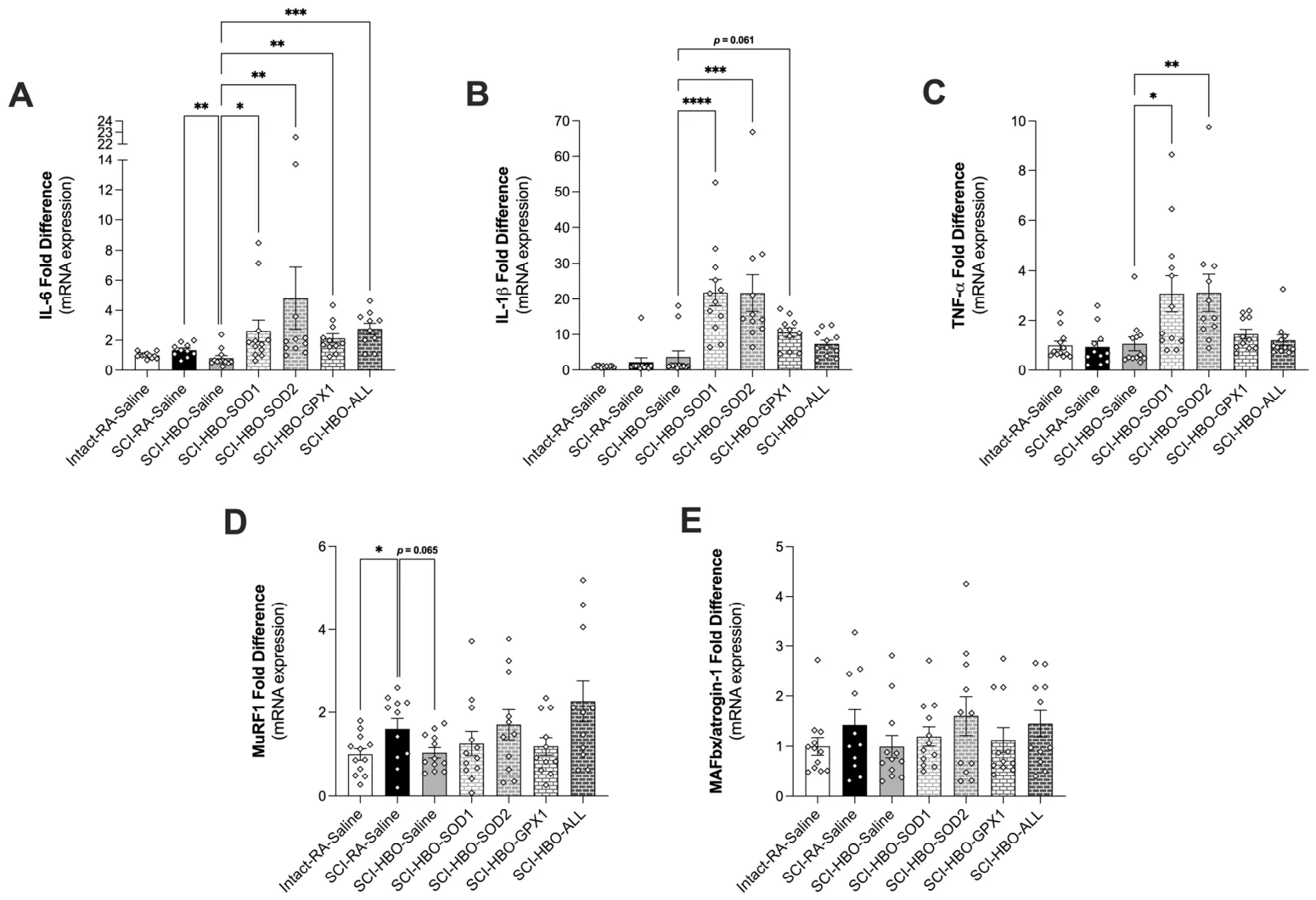
Diaphragm muscle mRNA expression of (**A**) IL-6; (**B**) IL-1β; (**C**) TNF-α; (**D**) MuRF1; and (**E**) MAFbx/atrogin-1. Open diamonds depict individual data points. Values are presented as mean ± SEM. * = *p* < 0.05, ** = *p* < 0.01, *** = *p* < 0.001 and **** = *p* < 0.0001. Group sample sizes: *n* = 12 (Intact-RA-Saline, SCI-HBO-Saline and SCI-HBO-SOD1); *n* = 11 (SCI-RA-Saline (*n* = 10 for IL-6), SCI-HBO-SOD2, SCI-HBO-GPX1 and SCI-HBO-ALL).

Diaphragm mRNA expression of the inflammatory markers IL-6, IL-1β and TNF-α showed a significant decrease in diaphragm IL-6 in the SCI-HBO-Saline group compared to SCI-RA-Saline (Figure 8A–C). All four ASO-treated groups had a significant increase in IL-6 gene expression compared to SCI-HBO-Saline. IL-1β and TNF-α were elevated in the SCI-HBO-SOD1 and SCI-HBO-SOD2 groups compared to SCI-HBO-Saline. Diaphragm gene expression of the atrophy gene MuRF1 showed a significant increase in the SCI-RA-Saline group compared to Intact-RA-Saline, with a trend for HBO treatment to attenuate this increase (*p* = 0.065) (Figure 8D). No difference existed between groups for MAFbx/atrogin-1 (Figure 8E).

### 3.7. Plasma Biomarkers

Measurement of plasma cytokine levels showed a reduction in leptin in both the SCI-RA-Saline and SCI-HBO-Saline groups compared to Intact-RA-Saline (Table 1). Compared to SCI-HBO-Saline, SCI-HBO-SOD1, SCI-HBO-SOD2, SCI-HBO-GPX1 and SCI-HBO-ALL had increased plasma macrophage inflammatory protein-1α (MIP-1α), IL-10, IL-18 and interferon-γ-induced protein 10 (IP-10). Plasma myokine levels showed a significant reduction in follistatin-like 1 (FSTL-1) and osteocrin compared to Intact-RA-Saline (Table 2). Erythropoietin (EPO) was greater in the SCI-HBO-GPX1 group compared to SCI-HBO-Saline and FGF21 was greater in the SCI-HBO-ALL group compared to SCI-HBO-Saline.

## 4. Discussion

Diaphragm muscle fiber atrophy and impaired contractility are primary consequences of cSCI, resulting from disruption of bulbospinal respiratory drive from the brainstem to the phrenic motor neurons and partial or complete respiratory muscle inactivity [1,2]. Due to its continuous activation to maintain pulmonary ventilation, airway clearance and postural stability, the diaphragm is highly sensitive to periods of inactivity and our previous work has shown reduced muscle fiber size and specific force production occur within 24–36 h following unilateral C2 hemisection injury [11,30]. In addition, these rapid effects are exacerbated when combined with mechanical ventilation, furthering respiratory impairment and greatly enhancing the risk of ventilator dependency [11]. While limited therapeutic options exist to improve respiratory function in people with cSCI, our recent work using a preclinical model of lateral cervical spinal contusion shows that daily HBO therapy initiated in the acute phase following injury is sufficient to improve diaphragm force production [15,16]. Our current work confirms these beneficial effects of HBO therapy and expands upon these findings to identify the underlying physiological mechanisms.

### 4.1. HBO Therapy and Muscle Antioxidant Expression

Skeletal muscle blood flow is reduced in response to inactivity, resulting in tissue ischemia, ROS production and muscle proteolysis [31,32,33]. In people with SCI, limb muscle atrophy occurs concomitant with reduced blood flow to paralyzed muscles, with loss of autonomic nervous system control a potential contributing factor to tissue hypoxia and muscle weakness [34,35]. In mechanically ventilated rodents, diaphragm inactivity also severely reduces tissue blood flow [31,33,36]. The proposed physiological mechanisms contributing to the diaphragm’s inability to appropriately adjust blood flow to meet oxygen demand with mechanical ventilation include the supraphysiological pressure applied to the thoracic cavity and the caudal displacement of the diaphragm [36,37,38]. Thus, the potential need for mechanical ventilation to sustain adequate ventilation may exacerbate the ischemic response to cSCI by further limiting the diaphragm’s ability to meet metabolic demand. Decreased tissue oxygen concentration can result in a proportional increase in mitochondrial ROS levels resulting from impaired electron transport activity, suppressed NADH and FADH_2_ oxidation and increased reduction in O_2_ to superoxide (O_2_^• –^) [39]. This impairment in oxidative phosphorylation can also result in a metabolic shift to glycolysis for ATP production, which reduces antioxidant capacity and promotes activation of autophagy and apoptotic cell death signaling [40]. In contrast, initiating HBO therapy in the acute phase following cSCI can increase cellular oxygen concentration, reduce oxidative stress and alter the adverse diaphragm response to muscle inactivity [15,16]. In both healthy and injured skeletal muscle, HBO exposure has been shown to affect antioxidant levels, with studies demonstrating increased expression of SOD1, SOD2 and GPX in response to both a single HBO exposure and chronic HBO treatment [15,41]. These studies and others support the use of HBO to reduce muscle injury through its positive effects on antioxidant capacity to maintain redox balance and promote muscle regrowth and/or prevent muscle proteolysis [15,41,42,43,44]. Importantly, work in this area has also identified the need to initiate HBO acutely following injury, with reports suggesting that HBO therapy is most effective at preserving skeletal muscle when initiated within the first 24 h following injury [45,46]. This supports current data demonstrating that early exposure to HBO post-SCI is critical to providing maximum benefit [47].

### 4.2. HBO Therapy and Respiratory Muscle Function

Studies investigating breathing recovery following cSCI have shown a transient deficit in quiet breathing followed by spontaneous recovery over the course of 10 days [16]. Our results agree with these previous findings as day 10 respiratory outcomes showed no significant differences between groups for respiratory rate, tidal volume or minute ventilation. This outcome may indicate the plethysmography was insufficient to detect any deficit or benefit due to robust spontaneous recovery and that identifying changes in these respiratory outcomes may require a more severe injury model or use of EMG muscle recordings to evaluate how breathing was accomplished. However, evaluation of individual breath inspiratory time, expiratory time and the I:E ratio revealed an effect of HBO to reduce expiratory time, resulting in an elevation of the I:E ratio. This shift in the respiratory cycle with HBO has the potential to improve oxygenation by increasing mean airway pressure and alveolar recruitment [48]. Interestingly, when compared to the SCI-HBO-Saline group, prevention of the HBO-induced increase in SOD1 mitigated the effect of HBO to increase the I:E ratio. This difference could potentially be related to elevated oxidative stress within the lungs or the medullary circuits. More work is needed to clearly define the importance of these subtle differences in respiration.

Direct evaluation of diaphragm muscle function supports previous work from our group demonstrating that 10 days of HBO is sufficient to improve diaphragm muscle contractility [15,16]. Additionally, our data reveal a requirement for both SOD1 and SOD2 for the HBO-induced improvement in diaphragm contractility at all stimulation frequencies tested and for GPX1 at frequencies of 1–60 Hz. However, the combined knockdown of SOD1, SOD2 and GPX1 did not result in a significant reduction in force production compared to the SCI-HBO-Saline group. While the mechanism for this is unclear, it is possible that alternative antioxidant defenses were upregulated in the diaphragm of these rats to prevent protein oxidation and muscle dysfunction. Antioxidant signaling is complex and reports have demonstrated mixed effects of single and double knockdown on muscle health and regulation of defensive pathways [21,49,50]. Examples of these divergent findings include no change in antioxidant expression [51], increased glutathione and SOD1 in response to SOD2 knockdown [21,49,50], increased ascorbate in response to GPX1 knockdown [52] and reduced GPX1 in response to SOD1 knockdown [53]. Thus, work is needed to fully elucidate the impact of SOD1, SOD2 and/or GPX1 knockdown on compensatory detoxification pathways and muscle function.

Interestingly, cSCI only resulted in atrophy of diaphragm muscle type I fibers, with no effect of any of the ASO knockdown groups to alter muscle fiber size. Further work is needed to understand the effects of cSCI and HBO on diaphragm muscle fiber size, as evaluation of diaphragm muscle fiber cross-sectional area has shown variable outcomes, with both atrophy of type I and type IIb/x fibers and no effect of cSCI demonstrated previously [15,16]. It is possible that differences in remaining diaphragm activity may account for some of the differences established across studies evaluating diaphragm atrophy and cSCI [54].

### 4.3. HBO Therapy and Diaphragm Muscle Proteolysis

Increased antioxidant expression can improve muscle health and recovery from injury by inhibiting tissue inflammation and preventing damage to and breakdown of muscle contractile proteins [55,56,57]. We have shown that antioxidant administration acutely following C2 hemisection improves diaphragm muscle force production and fiber size measured 36 h following injury [11]. This protective effect was associated with altered redox balance, inflammatory signaling and proteolytic signaling, which were also seen in the diaphragm following cSCI and 10 days of HBO therapy [11,15]. Interestingly, prevention of the HBO-induced increase in SOD1, SOD2 and/or GPX1 diminished these beneficial effects. Specifically, compared to SCI-HBO-Saline, knockdown of SOD2 and GPX1 resulted in an elevation in mitochondrial H_2_O_2_ emission, with no effect of knocking down SOD1, the cytosolic SOD isoform. In addition, all four ASO-treated groups had elevated mRNA levels of inflammatory genes, including IL-6, IL-1β and TNF-α. However, there was no effect of ASO treatment on MuRF1 or MAFbx/atrogin-1 E3 ligase gene expression compared to SCI-HBO-Saline.

Despite evidence demonstrating HBO therapy can suppress SCI-induced systemic pro-inflammatory signaling driven by NF-κB [17,58], our data did not show significant differences in plasma levels of circulating cytokines at 10 days following injury in HBO-treated compared to room air-treated cSCI rats. However, similar to diaphragm gene expression changes, knocking down SOD1, SOD2 and/or GPX1 antioxidant expression resulted in an increase in the circulating levels of the pro-inflammatory cytokines IL-18, IP-10 and MIP-1α. These cytokines are sensitive to cellular stress and redox balance, with evidence indicating an inverse relationship between antioxidant expression and IL-18, IP-10 and MIP-1α levels [59,60]. In addition, these cytokines have been shown to promote the recruitment of damaging immune cells to skeletal muscle, resulting in the disruption of metabolic signaling and activation of proteolytic pathways, leading to reduced muscle quality [61,62]. Additional work is needed to understand if the effect on plasma cytokines is related to the systemic effects of the ASOs, directly related to muscle-specific inflammatory signaling or potentially related to off-target knockdown linked to the use of ASOs in place of tissue-specific knockdown.

### 4.4. Limitations

This study provides evidence to support the hypothesis that HBO therapy initiated in the acute phase following cSCI diminishes diaphragm dysfunction, in part, through the upregulation of the antioxidant enzymes SOD1, SOD2 and GPX1. However, a limitation of this study is the exclusive use of male rats. While our previous publication did not reveal a sex difference in the diaphragm force-frequency response to cSCI or HBO therapy [16], data supporting the postulate that HBO induces an increase in SOD1, SOD2 and GPX1 were observed in male rats [15]. As this study did not confirm the HBO-induced increase in SOD1, SOD2 and GPX1 previously demonstrated, additional work is needed to verify the effect of HBO on the diaphragm expression of each antioxidant in male rats. Work is also needed in females to determine whether HBO induces an increase in SOD1, SOD2 and GPX1 expression in the diaphragm of cSCI rats and; if so, an identical study is needed to investigate the contribution of each antioxidant enzyme. Additional considerations in future studies should be made to include both room air, spinal intact and room air, cSCI groups receiving ASO-treatment to determine the effects of antioxidant knockdown on diaphragm function in healthy and injured rats, independent of HBO therapy. Also, although our measurements of inflammatory markers in this study do not suggest that the ASOs stimulated an immune response or that there was an ASO dose-dependent response, the use of the ASO vehicle rather than a scrambled ASO given at both 10 mg/kg/day and 30 mg/kg/day as a control for off-target and dose-dependent effects, is a limitation due to the potential for non-specific, sequence-dependent effects of phosphorothioate oligonucleotides. Interestingly, as our power analysis was performed using diaphragm-specific force as the most meaningful primary outcome measure and several statistical comparisons approached but did not reach statistical significance, it is possible that our sample size was not adequate to detect genuine effects for all dependent measures performed. Finally, the SCI-HBO-ALL group should be evaluated more critically in future studies to understand the potential contribution of compensatory antioxidant responses, biological interactions, non-specific effects and statistical power to the unexpected effect seen in this group.

## 5. Conclusions

HBO has been utilized in people with spinal injuries with therapy initiated as early as 24 h post-injury [63,64,65,66,67,68,69]. These studies have demonstrated the safety of HBO when used to treat these injuries and have also established the potential to provide neurological benefits. In preclinical studies utilizing a rat cervical contusion model, we have identified a dose–response where 3 ATA with 100% O_2_ provided the greatest benefit to respiratory muscle recovery compared to 2 ATA with 100% O_2_, 1 ATA with 100% O_2_ and 2 ATA with 10.5% O_2_ [16]. The current study provides the first mechanistic evidence supporting the contribution of increased antioxidant expression to improve diaphragm functional recovery following cSCI and HBO therapy. Our data indicate that reducing SOD1 and SOD2 had the greatest effect on muscle force production and inflammatory gene expression, potentially signifying greater muscle toxicity related to O_2_^•−^ formation compared to H_2_O_2_. However, the potential for alternate antioxidants like catalase to catalyze the reduction of H_2_O_2_ to H_2_O may account for the differences between these groups. While a potential confounding factor is the whole-body effect of ASO-induced gene silencing compared to a skeletal muscle-specific approach, this approach allowed for initial determination of the general requirement of systemic HBO-induced antioxidant expression to facilitate prevention of diaphragm dysfunction. However, selective blockade would be necessary in the future to understand the effect of increased spinal cord antioxidant expression compared to diaphragm-specific expression in HBO-treated rats undergoing cervical contusion injury. In addition, work is needed to understand the potential compensatory activity of other classes of antioxidant enzymes that support redox balance when antioxidant upregulation is prevented. Finally, these data support the need for continued investigation into the use of HBO as a therapeutic intervention for people with SCI and the use of preclinical evaluation to further understanding of the dose, timing and mechanisms of HBO-induced prevention of cSCI-related respiratory dysfunction.

## Figures and Tables

**Table 1:** Plasma cytokines and chemokines. A square root transformation was used for all plasma data. *N* = 11–12/group. Data is presented as mean ± SEM. ^§^ significantly different vs. Intact-RA-Saline (*p* < 0.05) and * significantly different vs. SCI-HBO-Saline (*p* < 0.05). Group sample sizes: *n* = 12 (SCI-HBO-Saline and SCI-HBO-SOD1) and *n* = 11 (Intact-RA-Saline, SCI-RA-Saline, SCI-HBO-SOD2, SCI-HBO-GPX1 and SCI-HBO-ALL). Plasma data underwent a square root transformation prior to analysis and is presented as (PG/ML)^1/2^.

(PG/ML)^1/2^	INTACT-RA-SALINE			SCI-RA-SALINE			SCI-HBO-SALINE			SCI-HBO-SOD1			SCI-HBO-SOD2			SCI-HBO-GPX1			SCI-HBO-ALL		
G-CSF	4.08	±	1.57	4.79	±	0.98	4.41	±	1.59	2.06	±	0.48	5.88	±	1.71	9.09	±	2.92	3.62	±	1.01
EOTAXIN	4.13	±	0.93	4.45	±	0.76	4.92	±	1.21	3.71	±	0.49	5.41	±	1.25	5.65	±	1.18	2.94	±	0.39
GM-CSF	7.53	±	1.05	10.88	±	1.95	9.33	±	1.93	8.04	±	0.87	8.48	±	1.82	11.96	±	2.62	5.99	±	0.61
IL-1α	7.38	±	2.91	7.92	±	1.97	8.76	±	3.75	4.26	±	1.13	9.51	±	2.99	9.97	±	3.04	4.15	±	1.07
LEPTIN	82.21	±	6.47	38.76	±	5.07 ^§^	35.43	±	1.63 ^§^	39.08	±	2.10	33.97	±	3.48	35.83	±	4.16	30.00	±	4.88
MIP-1α	1.66	±	0.60	2.21	±	0.48	1.19	±	0.16	4.26	±	0.41 *	3.63	±	0.43 *	3.30	±	0.35 *	4.68	±	0.62 *
IL-4	7.34	±	2.38	8.62	±	2.04	8.21	±	3.08	3.77	±	0.75	7.25	±	1.94	8.50	±	2.16	8.53	±	4.79
IL-1B	2.27	±	1.29	2.65	±	0.86	1.69	±	0.52	1.53	±	0.54	2.08	±	0.60	2.52	±	0.77	2.54	±	1.16
IL-2	6.05	±	2.45	7.85	±	2.01	3.46	±	0.51	3.21	±	0.22	4.70	±	1.62	7.30	±	2.34	3.40	±	0.40
IL-6	24.62	±	7.74	26.07	±	5.32	30.97	±	12.92	14.54	±	3.79	39.44	±	9.70	39.23	±	13.27	17.68	±	4.37
EGF	1.39	±	0.34	1.09	±	0.14	0.94	±	0.08	0.85	±	0.04	0.94	±	0.09	1.35	±	0.22	0.85	±	0.06
IL-13	4.49	±	1.41	4.52	±	0.88	5.36	±	2.08	3.29	±	0.76	6.01	±	1.72	6.55	±	2.08	2.23	±	0.69
IL-10	4.57	±	1.93	4.27	±	1.21	4.83	±	1.25	19.86	±	1.29 *	18.05	±	1.31 *	12.40	±	1.16 *	19.50	±	2.69 *
IL-12P70	7.15	±	2.25	11.51	±	3.45	6.08	±	2.22	3.48	±	0.28	8.30	±	2.53	8.18	±	2.45	4.05	±	0.79
IFN	10.76	±	3.55	10.31	±	2.25	11.55	±	5.13	7.07	±	0.90	10.69	±	3.41	11.29	±	3.45	5.22	±	1.10
IL-5	5.45	±	0.87	7.25	±	1.11	6.98	±	0.92	5.05	±	0.42	6.93	±	1.41	8.96	±	1.37	6.26	±	0.82
IL-17A	5.27	±	1.58	4.90	±	0.97	5.00	±	1.25	3.71	±	0.54	6.24	±	1.61	8.36	±	2.47	5.01	±	1.12
IL-18	4.92	±	2.08	6.39	±	1.56	2.71	±	0.28	4.44	±	0.32 *	4.95	±	1.21 *	6.18	±	1.39 *	5.08	±	0.57 *
MCP-1	14.34	±	2.32	15.26	±	2.09	18.74	±	6.03	21.02	±	2.88	21.21	±	3.59	17.85	±	1.79	11.53	±	1.92
IP-10	9.56	±	0.98	9.78	±	0.69	12.64	±	1.77	22.38	±	2.52 *	25.12	±	4.18 *	17.78	±	1.11 *	24.56	±	3.23 *
GRO/KC/CINC-1	10.59	±	3.13	9.15	±	2.51	12.12	±	5.26	8.43	±	2.15	7.38	±	2.25	11.46	±	2.75	4.03	±	1.25
VEGF	6.36	±	0.90	6.45	±	0.73	6.01	±	0.40	6.73	±	0.17	6.27	±	0.41	6.85	±	0.54	5.72	±	0.36
FRACTALKINE	4.86	±	0.84	4.40	±	0.68	3.91	±	0.32	3.59	±	0.16	3.94	±	0.31	3.93	±	0.43	3.70	±	0.36
LIX	10.84	±	1.86	10.32	±	1.44	11.94	±	1.89	8.03	±	0.63	12.44	±	3.52	12.62	±	2.47	6.75	±	1.64
MIP-2	5.88	±	0.85	6.46	±	1.18	5.81	±	1.68	4.28	±	0.69	5.61	±	1.15	6.24	±	1.16	3.43	±	0.62
TNFα	0.87	±	0.21	1.42	±	0.35	0.75	±	0.10	0.77	±	0.11	1.14	±	0.31	1.44	±	0.36	0.63	±	0.09
RANTES	24.58	±	2.45	18.42	±	1.39	20.06	±	1.98	18.04	±	1.27	20.60	±	2.84	20.09	±	2.27	16.33	±	1.68

**Table 2:** Plasma myokines. A square root transformation was used for all plasma data. *N* = 10–12/group. Data is presented as mean ± SEM. ^§^ significantly different vs. Intact-RA-Saline (*p* < 0.05) and * significantly different vs. SCI-HBO-Saline (*p* < 0.05). Group sample sizes: *n* = 12 (Intact-RA-Saline); *n* = 11 (SCI-HBO-SOD1 and SCI-HBO-GPX1); and *n* = 10 (SCI-RA-Saline, SCI-HBO-Saline, SCI-HBO-SOD2 and SCI-HBO-ALL). Plasma data underwent a square root transformation prior to analysis and is presented as (PG/ML)^1/2^.

(PG/ML)^1/2^	INTACT-RA-SALINE			SCI-RA-SALINE			SCI-HBO-SALINE			SCI-HBO-SOD1			SCI-HBO-SOD2			SCI-HBO-GPX1			SCI-HBO-ALL		
**BDNF**	5.10	±	1.12	4.71	±	1.24	4.17	±	0.74	2.32	±	0.47	4.07	±	1.25	4.43	±	1.72	3.20	±	0.74
**EPO**	3.70	±	0.48	4.76	±	0.90	3.40	±	0.74	5.52	±	1.04	4.67	±	0.47	6.78	±	0.83 *	6.04	±	0.93
**FGF21**	6.81	±	0.67	9.24	±	0.81	9.56	±	0.89	10.38	±	0.74	9.53	±	1.27	9.10	±	1.02	14.76	±	1.98 *
**FRACTALKINE**	4.36	±	0.21	4.00	±	0.29	4.11	±	0.33	3.70	±	0.31	3.79	±	0.32	3.54	±	0.31	4.31	±	0.39
**FSTL-1**	18.75	±	2.22	13.99	±	3.64	8.72	±	2.46 ^§^	11.61	±	2.34	8.69	±	2.50	13.06	±	4.14	14.61	±	3.14
**OSTEOCRIN**	7.67	±	0.37	6.76	±	0.44	5.56	±	0.50 ^§^	5.61	±	0.45	5.54	±	0.37	6.28	±	0.55	4.41	±	0.41
**SPARC**	1.00	±	0.04	0.98	±	0.14	1.02	±	0.06	0.78	±	0.07	0.78	±	0.08	0.88	±	0.15	1.10	±	0.14

## Data Availability

The original contributions presented in this study can be found at: https://doi.org/10.6084/m9.figshare.32588487.
